# Targeting of Mitochondria-Endoplasmic Reticulum by Fluorescent Macrocyclic Compounds

**DOI:** 10.1371/journal.pone.0027078

**Published:** 2011-11-21

**Authors:** Carla Cruz, Elisa Cairrao, Samuel Silvestre, Luiza Breitenfeld, Paulo Almeida, João A. Queiroz

**Affiliations:** CICS-UBI - Centro de Investigação em Ciências da Saúde, Universidade da Beira Interior, Covilhã, Portugal; Shantou University Medical College, China

## Abstract

**Background:**

Useful probes of the intracellular environment that target a specific organelle in order to allow direct observation of the changes in these regions is of high current interest. Macrocyclic ligands have already revealed themselves as important selective hosts in some biological applications, forming stable and specific complexes. Therefore, in this paper, several macrocyclic ligands are evaluated as potential molecular probes.

**Methodology:**

Four polyammonium macrocycles and one macrotricyclic bearing pyridine and phenanthroline chromophores have been synthesised and evaluated as molecular probes. The cytotoxicity of the compounds has been analyzed using human breast cancer cells (MCF-7), non-cancerous human dermal fibroblasts (NHDF) and human adult dermal skin fibroblasts from a breast cancer patient (P14). All the compounds showed low toxicity at concentrations ranging from 10 nM to 10 µM, except for [32]phen_2_N_4_ which proved to be highly cytotoxic for MCF-7 cells. Flow cytometry studies evidenced that the percentage of apoptotic and necrotic MCF-7 and NHDF cells induced by the compounds is considerably low. Also, flow cytometry analysis showed that some compounds seem to modify the mitochondrial membrane potential (MMP) of the cells. Fluorescence microscopy evidenced that compounds easily cross the plasma membrane (5 min) and accumulated into the mitochondria, as confirmed by co-localization with MitoTracker Green™. The fluorescence images also evidenced an intact mitochondria structure after 48 h. Moreover, reticular staining suggestive of endoplasmic reticulum (ER) localization, in addition to the mitochondrial one, has been found by confocal microscopy.

**Conclusion:**

Our study reveals that compounds Me_2_[28]py_2_N_6_, cryptphen, [16]phenN_2_, [30]phen_2_N_6_, have low toxicity and localize in mitochondria and ER. The ability of these compounds for translocating the cellular membrane (5 min) without special conditioning of the cells or derivatization of the probe, the time-dependent localization (48 h) and the cellular viability provide a proof-of-concept towards their use as promising probes towards biomedical studies.

## Introduction

The development of new responsive fluorescent probes that involve minimal perturbation of the biological system is essential for understanding the structure and the function of the cellular processes [1]. These systems must be adapted to the constraints imposed by the complex intracellular environment [2]. Some key issues must be addressed in devising a probe suitable to be used in living cells. For instance, the probe should be able to cross the outer lipid membrane at relatively fast rate and maintain the integrity and performance at cellular level, should exhibit an intracellular localization profile that is amenable for imaging microscopy observation, and should target a specific organelle keeping the cell viability, proliferation and membrane permeability, among others. The majority of probes that have been developed over the last years are based on emissive metal complexes [3], recombinant proteins [4], semiconductor nanoparticles, [5] (often termed quantum dots) and organic fluorophores with low molecular weight, such as, rhodamine, fluorescein, cyanine dyes and dipyrroylmethane [6]–[9]. However, the application of these probes is limited due to several properties, such as: low water and medium incubation solubility, pH range, optimal p*K*_a_ value, toxicity and cell penetration [10].

The dynamic contacts between the subcellular organelles are a common structural and functional feature affecting many cellular processes [11]. For instance, several studies suggested that the close contact between vesicular/tubular networks of mitochondria and the endoplasmic reticulum (ER) are crucial for the synthesis and intracellular transport of phospholipids, as well as, for intracellular Ca^2+^ homeostasis, controlling fundamental processes like motility and contraction, secretion, cell growth, proliferation and apoptosis [12]. It has been found that MAM (mitochondria associated membranes) and peptidic tethers keep mitochondria and ER in close contact. Immunofluorescence analysis has already confirmed and explained this juxtaposition between ER-mitochondria. Recent studies have shown, by electron tomography and immunofluorescence analysis, that this inter-organellar protein linkage between ER and mitochondria can be weakened (or strengthened) by rupture (or enforcement) of their networks [13]–[14].

Available fluorescent dyes known to target specific organelles, such as, LysoTracker™, ER-Tracker™ and MitoTracker™, allow the monitorization specific functions of the corresponding organelle and can be used in lower concentrations for any experimental approach [15]. For instance, the chloromethyl derivatives of the fluorescent cationic rosamine probes (MitoTracker™), currently used as mitochondrial probes, are lipophilic and cationic compounds that can accumulate electrophorethically into mitochondria in response to changes of mitochondrial membrane potential [16]. Basically, the reactive chloromethyl groups can form covalent bonds with SH groups mitochondrial proteins, avoiding their release even if mitochondria depolarize [16].

Other types of molecules that have been observed to localize on mitochondria are based on europium(III) and terbium(III) complexes of heptadentate ligands bearing azaxanthone or azathiaxanthone [17]–[19]. Co-staining experiments with these complexes revealed that merging from mitochondria to the lysosomes occurred only after considerably long incubation times (>4 h incubation) [20].

Macrocyclic ligands, such as cyclodextrins [21], calixarenes [22], crown ethers [22] and polyheterocycles with oxazole rings [23], have already revealed themselves as important selective hosts in some biological applications: drug solubilizers, drug stabilizers and anticancer agents [24]–[26].

**Figure 1:**
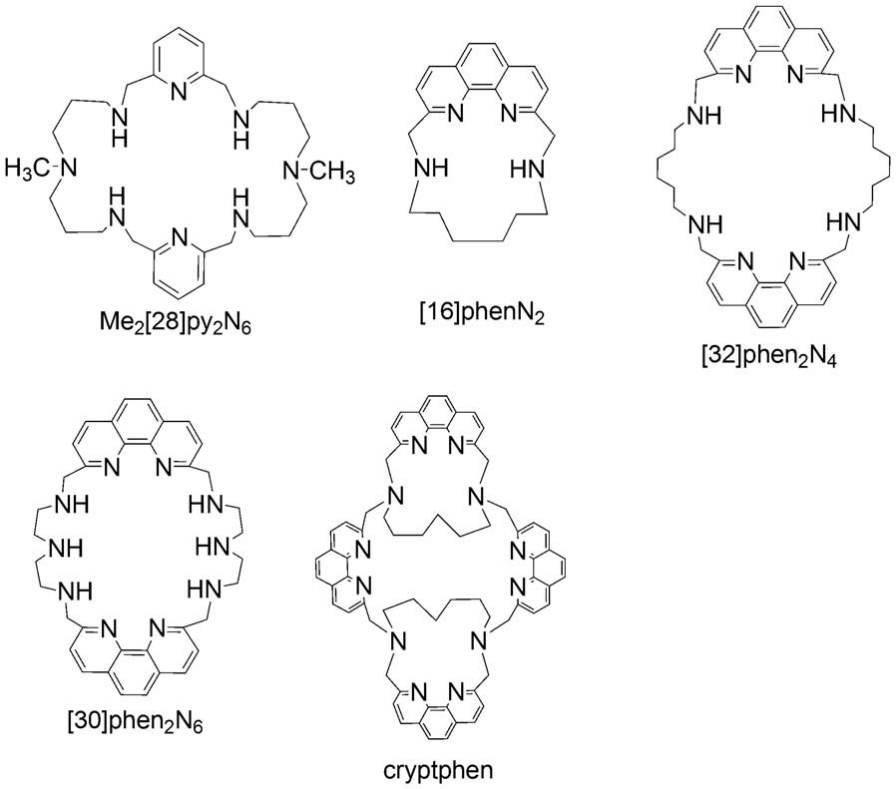
Chemical structures of pyridine or phenanthroline-containing macrocycles.

In this paper, the potential of five pyridine or phenanthroline-containing macrocycles (see Figure 1) as mitochondria and ER fluorescent probes is thoroughly explored. The cellular toxicity of the compounds is evaluated on breast cancer cells (MCF-7), non-cancerous human dermal fibroblasts (NHDF) and human adult dermal skin fibroblasts from a breast cancer patient (designated as P14) by MTT [3-(4,5-dimethylthiazol-2-yl)-2,5-diphenyltetrazolium bromide)] assay. The apoptosis induced on MCF-7 and NHDF cells and the MMP are evaluated by flow cytometry. The compounds ability to penetrate the intracellular medium and their localization is accomplished by fluorescence microscopy. Confocal images provide clear evidence of mitochondrial staining and additional ER localization.

## Results

### Ligand synthesis

Compounds Me_2_[28]py_2_N_6_, [32]phen_2_N_4_ and [30]phen_2_N_6_ have been prepared as recently described in [27], [28]. Compound [16]phenN_2_ is a versatile precursor for the assembly of macropolycyclic cryptphen, since it contains two secondary amine groups that can be connected by 2∶2 cyclizations with 1,0-phenanthroline moieties. The synthesis of [16]phenN_2_ and cryptphen is described in Experimental section, as well as, the compounds characterization.

### Toxicology assays on MCF-7, NHDF and P14 cells

**Figure 2:**
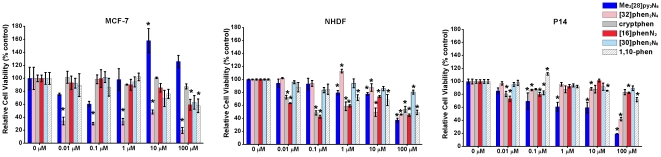
Relative cell viability of MCF-7, NHDF and P14 cells (0.5×10^4^ cells/well) incubated with Me_2_[28]py_2_N_6_, [32]phen_2_N_4_, cryptphen, [16]phenN_2_, [30]phen_2_N_6_ and 1,10-phen, in concentrations ranging from 0–100 µM and using the MTT assay at 48 h, determining formazan absorbance at 570 nm. Mean values ± SEM are obtained from three experimental determinations. Data are expressed as a percentage of cell viability in comparison with the control; the bars represent the mean and the lines represent the SEM associated. *P<0.05 versus the control (one-way ANOVA with Dunnet's post-hoc test).

One of the key issues to consider a probe suitable for living cells use is its cellular toxicity. In this context, the cytotoxicity of the five synthesised macrocyclic compounds is assessed by a 3-(4,5-dimethylthiazol-2-yl)-2,5-diphenyltetrazolium bromide (MTT) assay. The cytotoxicity of the starting material 1,10-phenanthroline-2,9-dicarbaldehyde is measured by MTT assay for comparison. In this quantitative colorimetric assay, the amount of MTT reduced by cells to its formazan derivative is quantified spectroscopically at 570 nm and is considered equivalent to the number of viable cells [29]. The cytotoxicity of the referred compounds is evaluated using human breast cancer cells (MCF-7), non-cancerous human dermal fibroblasts (NHDF) and human adult dermal skin fibroblasts (designated P14) obtained from the breast biopsy specimen of a woman diagnosed with breast cancer. All cells have been exposed to the six different compounds in five concentrations ranging from 10 nM to 100 µM during a period of exposition of 48 h. The results are presented in Figure 2.

Afterward, it has been analyzed if the compounds induced apoptotic or necrotic responses using flow cytometry. For this purpose, the fluorescein isothiocyanate (FITC)-conjugated annexin V - PI (propidium iodide) flow cytometry assay is employed, allowing the identification of apoptosis and necrosis [30]. Thus, MCF-7 and NHDF cells are treated with two concentrations (10 nM and 10 µM) of each compound for 48 h. These concentrations are above IC_50_ values, except for [32]phen_2_N_4_ on MCF-7 cells.

A dot-plot of Annexin V- FITC vs. PI showed four separate cell populations: viable cells (Annexin-V^−^/PI^−^), early apoptotic cells (Annexin-V^+^/PI^−^), late apoptotic cells (Annexin-V^+^/PI^+^) and necrotic cells (Annexin-V^−^/PI^+^).

**Figure 3:**
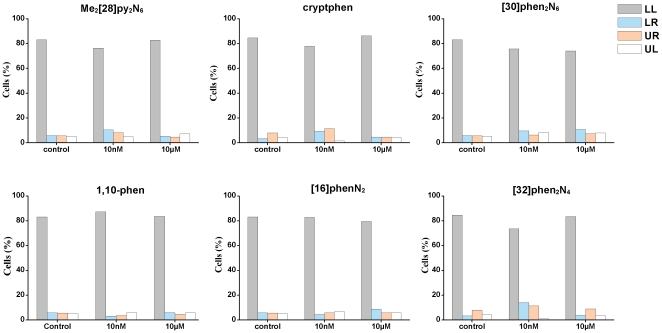
Relative percentages of viable cells (LL), early apoptotic cells (LR), necrotic cells (UL) and late apoptotic cells (UR) induced by Me_2_[28]py_2_N_6_, [32]phen_2_N_4_, cryptphen, [16]phenN_2_, [30]phen_2_N_6_ and 1,10-phen in NHDF cells. The cells have been treated with concentrations 10 nM and 10 µM of each compound for 48 h and comparing with the control.

**Figure 4:**
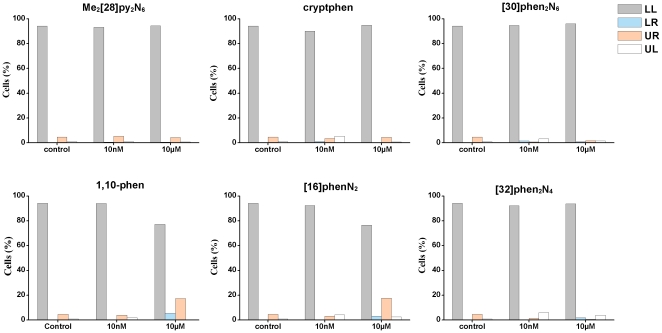
Relative percentages of viable cells (LL), early apoptotic cells (LR), necrotic cells (UL) and late apoptotic cells (UR) induced by Me_2_[28]py_2_N_6_, [32]phen_2_N_4_, cryptphen, [16]phenN_2_, [30]phen_2_N_6_ and 1,10-phen in MCF-7 cells. The cells have been treated with concentrations 10 nM and 10 µM of each compound for 48 h and comparing with the control.

Those percentages of NHDF and MCF-7cells that are in early apoptotis, late apoptotis and necrosis, comparing to the control, are presented in Figures 3 and 4, respectively.

Flow cytometry plots indicate that the percentage of early apoptotic and necrotic cells resulting from compounds treatment with NHDF is considerably low (<5%) at both concentrations (Figure 3). However, the percentage of late apoptotic NHDF cells was about 20% at the concentration of 10 µM for 1,10phen and [16]phenN_2_.

For MCF-7 cells the degree of early and late apoptosis induced by the majority of the compounds is less than 20% for both concentrations (10 nM and 10 µM). It seems that MCF-7 cells are more sensitive than NHDF cells (Figure 4).

### Microscopy studies

The timescale of trafficking of these compounds and their sub-cellular localization have been subsequently examined in MCF-7, NHDF and P14 cells using fluorescence microscopy. The cells are mounting on 0.1 mm thick glass cover slips by withdrawal of the respectively growth medium.

**Figure 5:**
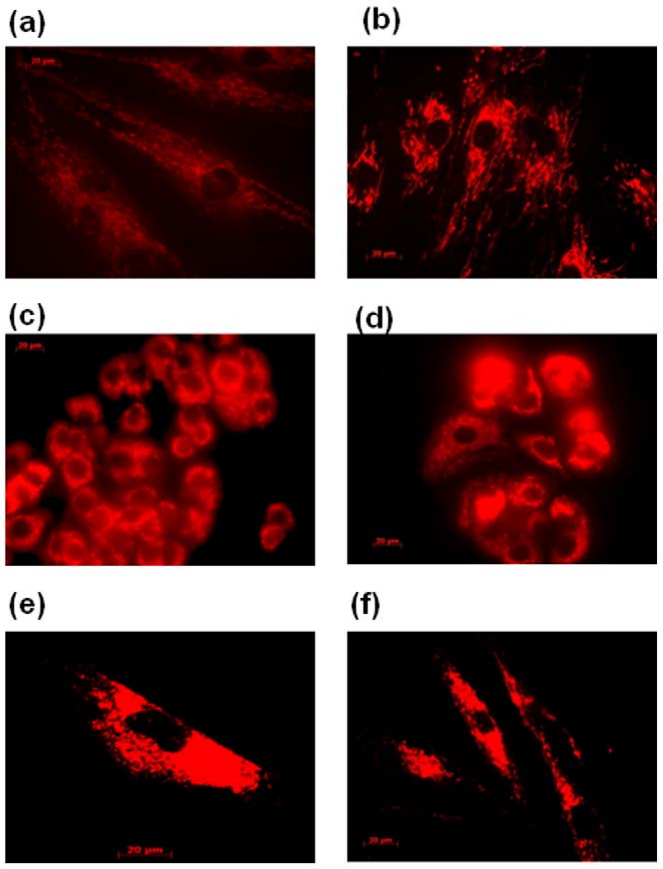
Fluorescence microscopy images of [30]phen_2_N_6_ (10 nM) in (a) NHDF, 5 min, (b) NHDF, 48 h, (c) MCF-7, 5 min, (d) MCF-7, 48 h, (e) P14, 5 min and (f) P14, 48 h; (1×10^4^ cells/dish).

**Figure 6:**
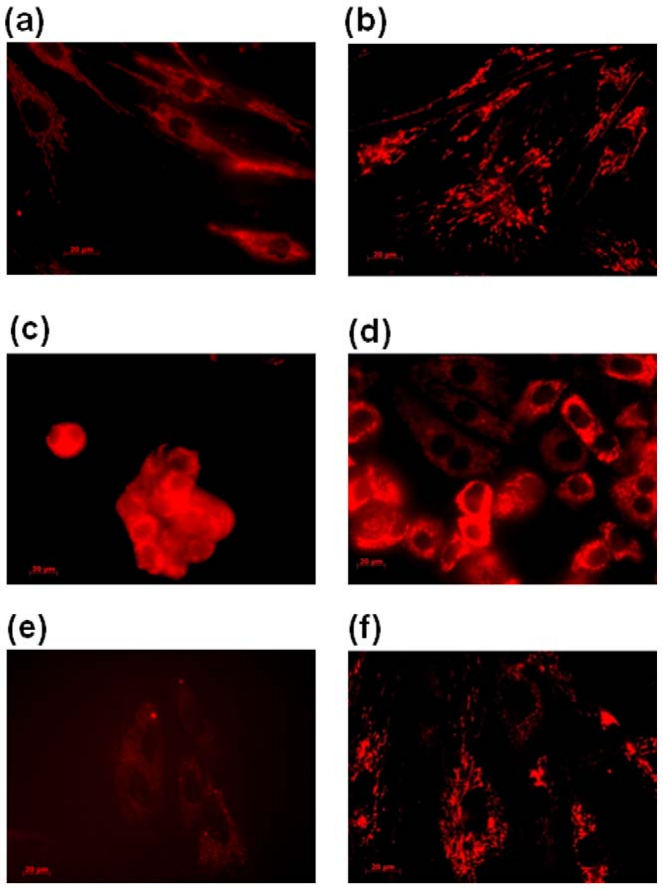
Fluorescence microscopy images of cryptphen (10 nM) in (a) NHDF, 5 min, (b) NHDF, 48 h, (c) MCF-7, 5 min, (d) MCF-7, 48 h, (e) P14, 5 min and (f) P14, 48 h; (1×10^4^ cells/dish).

The incubation times to test the ability compounds to penetrate into cells varied from 5 min to 48 h, and concentrations onto the cells were 10 nM and 100 µM by dissolving in appropriate medium. As a control measure, untreated cells were also examined and showed no fluorescence. By analyzing each image recorded, all the compounds in all cells rapidly crossed the cell membrane (5 min) and induced a slight stain in the first minutes of incubation, depending on the type of cells. However, for incubation times longer than 30 min, it seems that this difference in the stained disappeared, thus appearing that the compounds were localized into an organelle of the cytoplasm. The cells images at 5 min and 48 h of compounds [30]phen_2_N_6_ and cryptphen in MCF-7, NHDF and P14 cells are presented in Figures 5 and 6.

In Figures S1, S2, S3, S4 and S5 the fluorescence images of MCF-7, NHDF and P14 treated with cryptphen 10 nM after 1 h, 3 h, 6 h, 24 h and 36 h of incubation are presented. Based on these images, it has also been detected an intense fluorescence around the nuclear membrane, consistent with localization in mitochondria. Generally, no relevant signs of cytotoxicity have been observed when working at concentrations 10 nM and 100 µM over 48 h for all macrocycles, even in the presence of compound [32]phen_2_N_4_, which is clearly favorable for their use as cellular probes.

**Figure 7:**
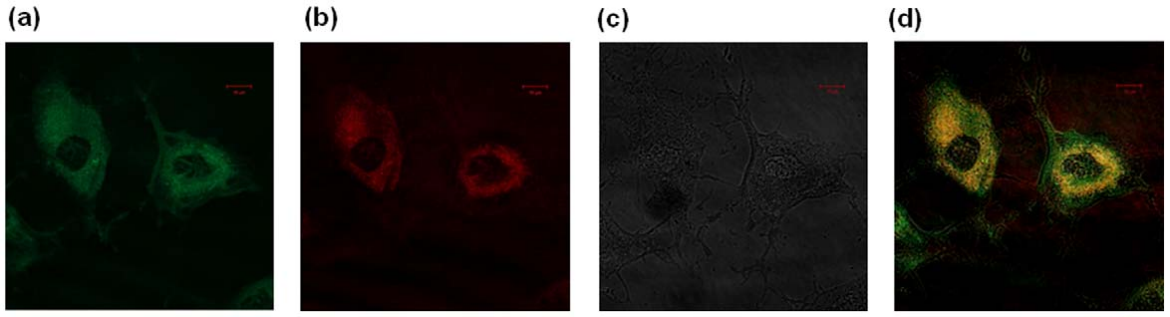
Distribution and co-localization of Me_2_[28]py_2_N_6_ (100 nM, incubation time 1 h) with MitoTracker Green™ (25 nM, incubation time 45 min) in NHDF cells (1×10^4^ cells/dish) collected with a confocal laser scanning microscope; fluorescence images of MitoTracker Green™ (a) and Me_2_[28]py_2_N_6_ (b); or differential interference contrast (c) and merged images of red and green channels (d); scale bar 10 microns.

**Figure 8:**
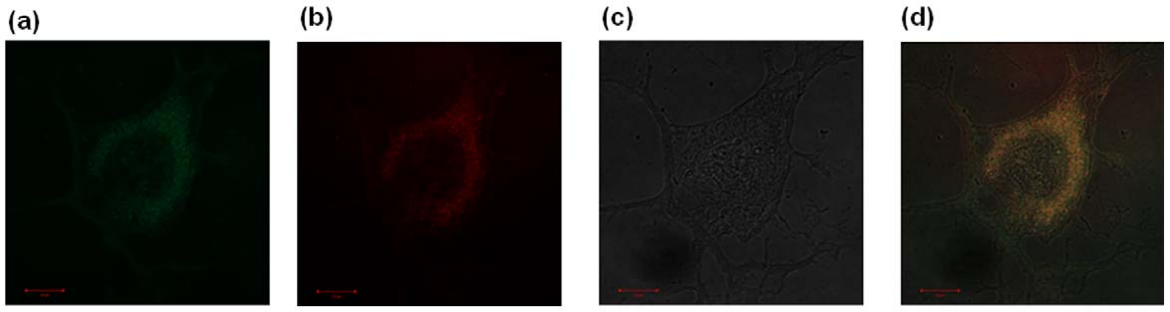
Distribution and co-localization of Me_2_[28]py_2_N_6_ (100 nM, incubation time 1 h) with ER-Tracker™ Green (100 nM, incubation time 30 min) in NHDF cells (1×10^4^ cells/dish) collected with a confocal laser scanning microscope; fluorescence images of ER-Tracker™ Green (a) and Me_2_[28]py_2_N_6_ (b); or differential interference contrast (c) and merged images of red and green channels (d); scale bar 10 microns.

Besides, the subcellular localization of the compounds has been confirmed by co-staining with MitoTracker™ (25 nM, 45 min of incubation) and ER-Tracker™ (100 nM, 30 min of incubation) probes in MCF-7, NHDF and P14 cells, with compound concentrations of 10 nM and 100 nM and for incubation times of 1 h. Also, co-staining with probe Hochest 33342 has shown that the compounds do not localize to the nucleus. Confocal fluorescence images showed that the luminescence pattern is consistent with a mitochondrial distribution, with the merged images of red and green channels showing good correspondence. In addition, partial reticular staining has been observed on confocal images, which is suggestive of endoplasmic reticulum localization. Selected images of Me_2_[28]py_2_N_6_ co-localization in NHDF cells with Mitotracker™ and ER-Tracker™ are presented in Figures 7 and 8, respectively.

## Discussion

The data presented in Figure 2 showed that, in spite of the general low cytotoxicity of the macrocycles, some interesting differences in their effects are noticeable on these three different human cells. Hence, compounds Me_2_[28]py_2_N_6_, cryptphen, [16]phenN_2_, [30]phen_2_N_6_, and 1,10-phen are not significantly cytotoxic to MCF-7 cells, according to the IC_50_ values that are higher than 100 µM. In contrast, the IC_50_ value for [32]phen_2_N_4_ is in low micromolar range (<0.01 µM), suggesting that at this concentration this compound shows a remarkable cytotoxicity. Otherwise, the data of [32]phen_2_N_4_ to fibroblast cells (NHDF and P14) suggested less cell toxicity. The IC_50_s are 78.92±2.82 and 85.30±1.79 µM in the NHDF and P14 cells, respectively. This toxic effect in cancer cells *versus* cell viability in normal cells encouraged us to further explore the interest of this compound as a potential future anticancer agent.

For the remaining compounds the IC_50_s obtained are in high micromolar range (>100 µM), except for Me_2_[28]py_2_N_6_ in P14 that has a IC_50_ of 36.79±4.68 µM, showing that Me_2_[28]py_2_N_6_ is more toxic to P14 than to NHDF cells (IC_50_ of 69.67±3.02 µM).

Also, at 0.1 µM for cryptphen and [16]phenN_2_ in NHDF cells, approximately 50% reduction in cell viability has been observed while in P14 it was not observed. However, the increase of the concentrations of these compounds to 1 µM, 10 µM and 100 µM does not lead to an increase in the NHDF and P14 cells death.

Hence, the cytotoxicity data obtained over 48 h cell incubation period at range of concentrations of 0.1 and 10 µM motivated us to further explore the potential utility of the fluorescent macrocycles cryptphen, [16]phenN_2_ and [30]phen_2_N_6_ as molecular probes.

Based on Annexin V- FITC vs. PI flow cytometry assay no remarkable effects of these compounds in the induction of apoptosis and necrosis has been observed in comparison with control cells. It has been found that MCF-7, as cancer cells, seems to be more sensitive at the concentrations tested than NHDF. Indeed, the higher cell viability value of MCF-7 incubated with 10 µM of Me_2_[28]py_2_N_6_, determined by MTT assay, can be an artifact since no relevant apoptosis has been found.

The sub-cellular localization of compounds was confirmed by the use of commercially available cellular stains for mitochondria, nucleus and endoplasmic reticulum. All the compounds studied do not localize to the nucleus. The inspection of the fluorescence microscopy images showed ER–mitochondria localization at all incubation times, from 5 min to 48 h. No signs of rupture of plasma membrane have been observed and the cellular architecture and integrity of the mitochondria was preserved in the presence of the compounds. The images showed the typical pattern of mitochondrial organization present in MCF-7 and NHDF obtained in previous studies [31]–[33]. Considering the nature of the chromophore structure (phenanthroline and pyridine) on the compounds localization, no marked changes have been observed on their mitochondrial-ER distribution in the three different human cells.

A possible explanation about their preferential accumulation in mitochondria is that at physiological pH the compounds carried out a delocalized positive charge, allowing permeating plasma membrane, as well as, the mitochondrial membranes [34].

In order to study the effects of the compounds in mitochondrial membrane potential (MMP) of MCF-7 and NHDF cells, flow cytometry assay was employed to monitor any fluorescence changes in MitoTracker Green™ and red fluorescence of chloromethyl-X-rosamine (CMXRos), treatment with the uncoupler 2,4-dinitrophenol (2,4-DNP) to collapse the MMP. While MitoTracker Green™ staining was relatively unaffected by changes in MMP, the CMXRos staining of mitochondria is sensitive to MMP [30]. Also, laser scanning confocal microscopy was employed to analyze changes in mitochondrial localization by treated cells with uncoupler 2,4-DNP and staining with MitoTracker Green™ and compounds.

The flow cytometry assay for MCF-7 showed that the compounds exhibit a decrease of red fluorescence comparing with the positive control (2,4-DNP), in particular for Me_2_[28]py_2_N_6_ and [16]phenN_2_. These changes in the MMP give rise to FL4/FL1 intensities lower than positive control. This seems to indicate that all the compounds disrupt the mitochondrial membrane potential of MCF-7 cells. Nevertheless, for NHDF cells only compounds [16]phenN_2_ and cryptphen seem to modify the MMP by exhibiting a decrease of FL4/FL1 ratio comparing to the positive control. For the remaining compounds no changes in the MMP was detected.

On the other hand, these changes do not seem to have a remarkable effect on cell death, according to the results of Annexin V- FITC presented in Figures 3 and 4.

The fluorescence images of the cells treated with 300 µM of uncoupler 2,4-DNP and stained with MitoTracker Green™ and compounds do not show significant differences in the co-localization in presence of 2,4-DNP. These compounds appear to localize mitochondria besides disrupting its membrane potential, as indicated by flow cytometry assay with CMXRos. The single laser scanning confocal microscopy images of MCF-7 cells stained with [30]phen_2_N_6_ and MitoTracker Green™ and treated with 2,4-DNP are presented in Figure S6. The untreated control cells are presented in Figure S7, for comparison.

Relatively to the additional reticular staining localization, this could be explained by spatial and functional organized network mediated by mitochondrial proteins and mitochondria-associated membranes that interconnect ER–mitochondria [14]. The molecular mechanisms that controlled this interaction require further investigation, which could be the subject of a future work.

In summary, our results clearly demonstrated that compounds Me_2_[28]py_2_N_6_, cryptphen, [16]phenN_2_, and [30]phen_2_N_6_, are able to be used as sub-cellular probes, having the ability for translocating the cellular membrane without a special conditioning of the cells, being also non-toxic at the concentrations used on the three human cells tested (MCF-7, NHDF and P14). In every case, all the compounds exhibited identical sub-cellular localization patterns on mitochondria and endoplasmic reticulum in less than 1 h. This result also indicates that the nature of the chromophore (phenanthroline and pyridine) does not affect the cellular distribution. The fact that the compounds localize in both mitochondria and endoplasmic reticulum also makes them ideal candidates for their use as specific cellular probes, due to the intimate liaison of the endoplasmic reticulum–mitochondria. However, the compounds cell uptake mechanism requires future investigation. The timescale of trafficking of these compounds (5 min), the time-dependent localization (48 h) and the cellular viability makes them promising molecular probes.

## Materials and Methods

Reagents and solvents were of the purest grade available, and were generally used without further treatment. 1,6-Hexanediamine, diethylenetriamine, N,N-bis-(3-aminopropyl)-methylamine and sodium borohydride were purchased from Aldrich. 1,10-phenanthroline-2,9 dicarboxaldehyde and 2,9-bis(bromomethyl)-1,10-phenanthroline were prepared as previously described in [35]. The synthesis of the compounds Me_2_[28]py_2_N_6_, [32]phen_2_N_4_ and [30]phen_2_N_6_ was also described previously [27], [28].

Elemental analyses and electron spray ionization (ESI) mass spectra were performed by the microanalysis service on a Fisons EA-1108 microanalyzer and on a Bruker Daltonics Apex-Qe instrument, respectively.

NMR spectra were recorded on a Bruker Avance II 400 operating at 400.13 MHz for protons and 100.62 MHz for carbons and/or a Bruker Avance III 600 spectrometer operating at 600.13 MHz for protons and 150.96 MHz for carbons, equipped with pulse gradient units. The reference used for the ^1^H NMR spectra in D_2_O and MeOD-*d*_4_ was 3-(trimethylsilyl)propanoic acid-*d*_4_ sodium salt. For ^13^C NMR spectra, dioxane was used as internal reference.

All spectra were acquired at temperature of 298 K and using the standard Bruker software.

### Me_2_[28]py_2_N_6_

Yield 85%. m.p. 206–207°C. ^1^H NMR (600 MHz, D_2_O): δ (ppm) 2.04 (8 H, qt, NCH_2_C*H*_2_CH_2_N), 2.58 (6 H, s, NC*H*_3_), 3.08 (16 H, m, NC*H*_2_CH_2_C*H*_2_N), 4.28 (8 H, s, pyC*H*_2_N), 7.45 (4 H, d, py) and 7.98 (2 H, t, py). ^13^C NMR (600 MHz, D_2_O): δ (ppm) 21.52 (NCH_2_ *C*H_2_CH_2_N), 40.79 (N*C*H_2_CH_2_CH_2_N), 40.09 (N*C*H_3_), 50.54 (NCH_2_CH_2_ *C*H_2_N), 52.89 (py*C*H_2_N), 123.76 (py), 139.92 (py) and 150.08 (py). ESI-MS: m/z: 497.52 [L+H]^+^. Anal. calc. for C_28_H_54_Cl_6_N_8_O_3_: % C 40.04, H 7.92, N 14.65; found: % C 40.22, H 7.86, N 14.60.

### [32]phen_2_N_4_

Yield 70%. m.p. 204–205°C. ^1^H NMR (600 MHz, D_2_O): δ (ppm) 1.45 (m, 8H, NCH_2_CH_2_C*H*_2_), 1.81 (m, 8H, NCH_2_C*H*_2_CH_2_), 3.20 (m, 8H, NC*H*_2_CH_2_CH_2_), 4.71 (s, 8H, phenC*H*_2_N), 7.81 (d, 4H, phen), 8.01 (s, 4H, phen) and 8.55 (d, 4H, phen); ^13^C NMR (600 MHz, D_2_O): δ (ppm) 25.59 (NCH_2_CH_2_ *C*H_2_), 27.08 (NCH_2_ *C*H_2_CH_2_), 48.89 (N*C*H_2_CH_2_CH_2_), 50.65 (phen*C*H_2_N), 123.97, 128.21, 130.55, 141.25,145.22 and 153.13 (phen). ESI-MS: *m/z*: 641.41 [L+H]^+^. Anal. calc. for C_40_H_65_Cl_5_N_8_O_6_: C 51.59, H 7.04, N 12.23; found: C 51.32, H 7.17, N 12.28.

### [30]phen_2_N_6_

Yield 40%. m.p. 226–227°C. ^1^H NMR (600 MHz, D_2_O): δ (ppm) 3.51 (t, 8H, NCH_2_C*H*_2_N), 3.62 (t, 8H, NC*H*_2_CH_2_N), 4.54 (s, 8H, phenC*H*_2_N), 7.63 (d, 4H, phen), 7.88 (s, 4H, phen), 8.35 (d, 4H, phen); ^13^C NMR (600 MHz, D_2_O): δ (ppm) 45.92 (NCH_2_ *C*H_2_N), 49.52 (N*C*H_2_CH_2_N), 56.57 (phen*C*H_2_N), 125.52, 129.78, 131.35, 142.07, 146.02, 153.45 (phen). ESI-MS: *m/z*: 615.37 [L+H]^+^. Anal. calc. for C_36_H_54_Cl_8_N_10_O_2_: % C 45.88, H 5.77, N 14.86; found: % C 45.75, H 6.09, N 14.63.

### [16]phenN_2_

1,10-phenanthroline-2,9-dicarboxaldehyde (0.22 g, 0.93 mmol) was previously dissolved in 100 mL of warm methanol and then cooled to room temperature. This solution was dropped into 250 mL methanol solution containing 1,6-hexanediamine (0.12 g, 1.03 mmol) during 6 h at room temperature. The resulting mixture was stirred for one night. The white solid formed was filtered off and dissolved in ethanol (30 mL). Sodium borohydride (0.09 g, 2.3 mmol) was then added in small portions to the cooled mixture (in ice bath) and left it under stirring for 12 h at room temperature. The solvent was removed under reduced pressure and the resulted residue was treated with water and repeatedly extracted with chloroform (4×30 mL). After extraction, the organic phases were combined and completely evaporated under vacuum and then dissolved in ethanol (20 mL). The pure compound was precipitated from concentrate hydrochloric acid solution as a white powder easy to filter upon 12 h at 4°C.

Yield 92%. m.p. 210–211°C. ^1^H NMR (600 MHz, D_2_O): δ (ppm) 1.44 (m, 4H, NCH_2_CH_2_C*H*_2_), 1.80 (m, 4H, NCH_2_C*H*_2_CH_2_), 3.22 (m, 4H,NC*H*_2_CH_2_CH_2_), 4.01 (s, 4H, phenC*H*_2_N), 7.76 (d, 2H, phen), 7.97 (s, 2H, phen) and 8.50 (d, 2H, phen); ^13^C NMR (600 MHz, D_2_O): δ (ppm) 24.32 (NCH_2_CH_2_ *C*H_2_), 32.90 (NCH_2_ *C*H_2_CH_2_), 49.90 (N*C*H_2_CH_2_CH_2_), 55.50 (phen*C*H_2_N), 122.05, 126.82, 128.11, 135.60, 144.32 and 159.80 (phen). ESI-MS: *m/z*: 321.21 [L+H]^+^. Anal. calc. for C_20_H_31_Cl_3_N_4_O_2_: % C 51.57, H 6.71, N 12.03; found: % C 52.05, H 7.09, N 12.03.

### cryptphen

A solution of [16]phenN_2_ (0.33 g, 0.83 mmol) in dry acetonitrile (30 mL) was added, over a period of 3 h to a refluxing and vigorously stirred suspension of 2,9-bis(bromomethyl)-1,10-phenanthroline (0.30 g, 0.83 mmol) and Na_2_CO_3_ (0.87 g, 8 mmol) in dry acetonitrile (40 mL). After the addition was completed the solution was refluxed for additional 24 h. The resulting suspension was filtered out and the solution was vacuum evaporated to give a oil. The product was chromatographed on neutral alumina with chloroform. The eluted fractions were collected and vacuum evaporated and then dissolved in ethanol (20 mL). The pure compound was precipitated from concentrate hydrobromic acid solution as a white powder.

Yield 31%. m.p. 220–221°C. ^1^H NMR (600 MHz, MeOD-*d*_4_): δ (ppm) 1.54 (m, 8H, NCH_2_CH_2_C*H*_2_), 1.90 (m, 8H, NCH_2_C*H*_2_CH_2_), 3.33 (m, 8H,NC*H*_2_CH_2_CH_2_), 4.75 (s, 8H, phenC*H*_2_N), 7.86 (d, 8H, phen), 8.04 (s, 8H, phen) and 8.59 (d, 8H, phen); ^13^C NMR (600 MHz, MeOD-*d*_4_): δ (ppm) 24.44 (NCH_2_CH_2_ *C*H_2_), 25.67 (NCH_2_ *C*H_2_CH_2_), 58.53 (N*C*H_2_CH_2_CH_2_), 57.44 (N*C*H_2_CH_2_CH_2_), 60.52 (phen*C*H_2_N), 59.90 (phen*C*H_2_N), 138.76, 139.63, 142.95, 149.48, 150.27 and 152.06 (phen). ESI-MS: *m/z*: 1049.54 [L+H]^+^. Anal. calc. for C_68_H_74_Br_8_N_12_O:% C 47.63, H 4.35, N 9.80; found: % C 47.97, H 4.39, N 9.96.

### *In vitro* studies using MCF-7, NHDF and P14 cells

The cells used in this study were human breast cancer (MCF-7), normal human dermal fibroblasts (NHDF) (both acquired to ATCC – American Type Culture Collection) and human adult dermal skin fibroblasts, designated P14, established from the breast biopsy specimen of a consenting post-menopausal woman diagnosed with breast cancer. We have obtained the ethics approval for our study from the ethics committee (Comissão de Ética, in Portuguese) at the Hospital Center of Cova da Beira (Centro Hospitalar da Cova da Beira, in Portuguese) in Covilhã, Portugal, regarding the full usage of Human Brest Cancer (Cancro da Mama, in Portuguese) Cells. The consent was in written form.

Unless otherwise stated, chemicals (analytical grade), assay reagents, culture media and supplements are all from Sigma-Aldrich. Experiments were performed in 48-well tissue culture plates and thick glass cover slips. The studied compounds were dissolved in DMSO (1,10-phenanthroline-2,9 dicarboxaldehyde), phosphate buffer (Me_2_[28]py_2_N_6_), DMSO/methanol 2∶1 ([32]phen_2_N_4_ and [16]phenN_2_) and 1∶2 (cryptphen) and phosphate buffer/methanol 1∶2 ([30]phen_2_N_6_). The final solvent concentration in the wells was ≤1%. This concentration of the solvents has no effect on cell viability (data not shown).

### Culture of cells

Cells were routinely maintained at 37°C in a humidified atmosphere containing 5% CO_2_. Human dermal fibroblasts were cultured in RPMI medium supplemented with 10% fetal bovine serum (FBS), HEPES (0.01 M), *L*-glutamine (0.02 M) and sodium pyruvate (0.001 M) and 1% antibiotic/antimycotic (10,000 units/mL penicillin, 10 mg/mL streptomycin and 25 µg/mL amphotericin B). Dubelco's Modified Eagle's Medium high glucose supplemented with 10% fetal bovine serum and 1% antibiotic/antimycotic was used to culture MCF-7 cells. All the cells were used between passages 3 to 10 in the experiments.

### Analysis of cell viability (Cytotoxicity assay - MTT test)

Cell viability was studied quantifying the extent of the reduction of 3-(4,5-dimethylthiazol-2-yl)-2,5-diphenyltetrazolium bromide (MTT) according to a previously described procedure [36]. Briefly, cells were seeded in 48-well plates (0.5×10^4^ cells/well) in the culture medium containing FBS and after 48 h they were treated with five concentrations (10 nM and 100 nM, 1 µM, 10 µM and 100 µM) of the different compounds for 48 h, with untreated cells serving as control. At the end of incubation the media in wells were removed and replaced with fresh media and MTT solution and incubated at 37°C for 4 h. Thereafter, media-containing MTT were removed and formazan crystals were dissolved and absorbance was recorded in Biorad 550 microplate reader at 570 nm. The extent of cell death was expressed as the percentage of cell viability in comparison with control cells.

### Fluorescence Microscopy

After growth in the glass bottom culture dishes coated with collagen (1×10^4^ cells/dish), the cells were incubated in the dark at 37°C with 10 nM of all the compounds and analyzed from 5 min to 48 h.

Prior to visualization, the excess probe was washed off by rinsing in PBS four times. After mounting these samples, the compounds were localized using a Zeiss AX10 microscope and the Axio Vision Real 4.6 software.

In the co-localization experiment, cells were incubated in the dark at 37°C with 100 nM of all the compounds for 1 h. The medium was removed and the cells were then treated for 1 h with 25 nM MitoTracker Green™, a potential-sensitive dye that accumulates in mitochondria or with ER-Tracker™ Green, a highly selective dye for the endoplasmic reticulum. Prior to visualization, excess probe was washed off by rinsing in PBS four times.

Confocal fluorescence imaging: the cells were observed with a confocal laser scanning microscope (Carl Zeiss, LSM510 Meta) with 405- and 488-nm laser excitation for the 4,6-diamidino-2-phenylindole (DAPI) and fluorescein isothiocyanate (FITC) channels, respectively. An appropriate emission band was selected for the two fluorescent channels. A 63× ApoChromat,1.4-NA objective (Carl Zeiss) was used to ensure high-resolution images. DIC images were collected simultaneously with transmitted light by excitation at 633 nm. Sequential rather than simultaneous acquisition was used to avoid bleed-through between the two fluorescent channels. Images were processed with LSM Image Browser (Zeiss), ImageJ and Adobe Photoshop.

### Flow Cytometry

To detect apoptosis, cells were stained with propidium iodide (PI) and fluorescein isothiocyanate (FITC)-conjugated annexin V using an Annexin V-FITC Apoptosis Detection Kit (BD PharmingenTM). Briefly, cells were seeded in 75 cm^3^ flasks in the culture medium containing FBS and after reaching approximated 90% confluence they were treated with two concentrations (10 nM and 10 µM) of each compound for 48 h, with untreated cells serving as control. At the end of incubation, cells were harvested by trypsin-EDTA treatment, washed twice with cold PBS and resuspended with 1× binding buffer (1 mL). Then, 100 µl of the cell suspension was transferred to a culture tube and annexin V-FITC (5 µl) and PI (10 µl) were added followed by incubation at room temperature in the dark for 15 min. After the addition of 400 ml of 1× binding buffer, stained cells were subjected to fluorescence-activated cell sorter (FACS) analysis using a FACSCalibur flow cytometer. The FITC fluorescence was measured between 515 and 545 nm and the PI fluorescence between 564 and 606 nm. Data analysis was performed using CellQuest™ Pro Software.

The assessment of mitochondrial membrane potential was determined as described previously [30]. Briefly, the cells were incubated with 200 nM of MitoTracker Green™ and 200 nM of Mitotracker Red (CMXRos) for 30 min prior to harvesting. The 2,4-dinitrophenol (DNP) was used as a mitochondrial uncoupler (300 µM, 2 h). The cells were treated with compounds concentration 10 µM for 48 h. The relative mitochondrial membrane potential was analyzed by flow cytometry by measuring the mean intensity of FL1 (green) and FL4 (red) channels. The FL4/FL1 ratio intensities of compounds treated and positive control (DNP) were compared and indicated if the compounds change the mitochondrial membrane potential of the cells.

### Statistics

All experiments were performed in triplicate. The IC_50_ values were calculated from the obtained dose-response curve by sigmoid fitting. Graphical results of citotoxicity were expressed as the average values ± SEM (standard error of mean). Comparison among multiple groups was analyzed by using a one-way ANOVA followed by Dunnet's post hoc tests to determine significant differences among the means. Difference between groups was considered statistically significant at P<0.05.

## Supporting Information

Figure S1 Fluorescence microscopy images of cryptphen in (a) NHDF, (b) MCF-7 and (c) P14; cells incubation with 10 nM of cryptphen during 1 h.
(TIF)

Figure S2 Fluorescence microscopy images of cryptphen in (a) NHDF, (b) MCF-7 and (c) P14; cells incubation with 10 nM of cryptphen during 3 h.
(TIF)

Figure S3 Fluorescence microscopy images of cryptphen in (a) NHDF, (b) MCF-7 and (c) P14; cells incubation with 10 nM of cryptphen during 6 h.
(TIF)

Figure S4 Fluorescence microscopy images of cryptphen in (a) NHDF, (b) MCF-7 and (c) P14; cells incubation with 10 nM of cryptphen during 24 h.
(TIF)

Figure S5 Fluorescence microscopy images of cryptphen in (a) NHDF, (b) MCF-7 and (c) P14; cells incubation with 10 nM of cryptphen during 36 h.
(TIF)

Figure S6 Single laser scanning confocal microscopy images of MCF-7 cells (1×10^4^ cells/dish) treated with 2,4-DNP (300 µM, 2 h) and stained with [30]phen_2_N_6_ (100 nM, 1 h) and MitoTracker Green™ (25 nM, incubation time 30 min); (a) fluorescence images of MitoTracker Green™, (b) [30]phen_2_N_6_, (c) differential interference contrast (d) and merged images of red and green channels; scale bar 10 microns.
(TIF)

Figure S7 Single laser scanning confocal microscopy images of MCF-7 cells (1×10^4^ cells/dish) stained with [30]phen_2_N_6_ (100 nM, 1 h) and MitoTracker Green™ (25 nM, incubation time 30 min) without 2,4-DNP; (a) fluorescence images of MitoTracker Green™, (b) [30]phen_2_N_6_, (c) differential interference contrast (d) and merged images of red and green channels; scale bar 10 microns.
(TIF)
