# Supplementary figures and images for: Targeting of Mitochondria-Endoplasmic Reticulum by Fluorescent Macrocyclic Compounds
Source: PLoS One. 2011 Nov 21;6(11):e27078. doi: 10.1371/journal.pone.0027078 (PMC3221659; doi:10.1371/journal.pone.0027078)

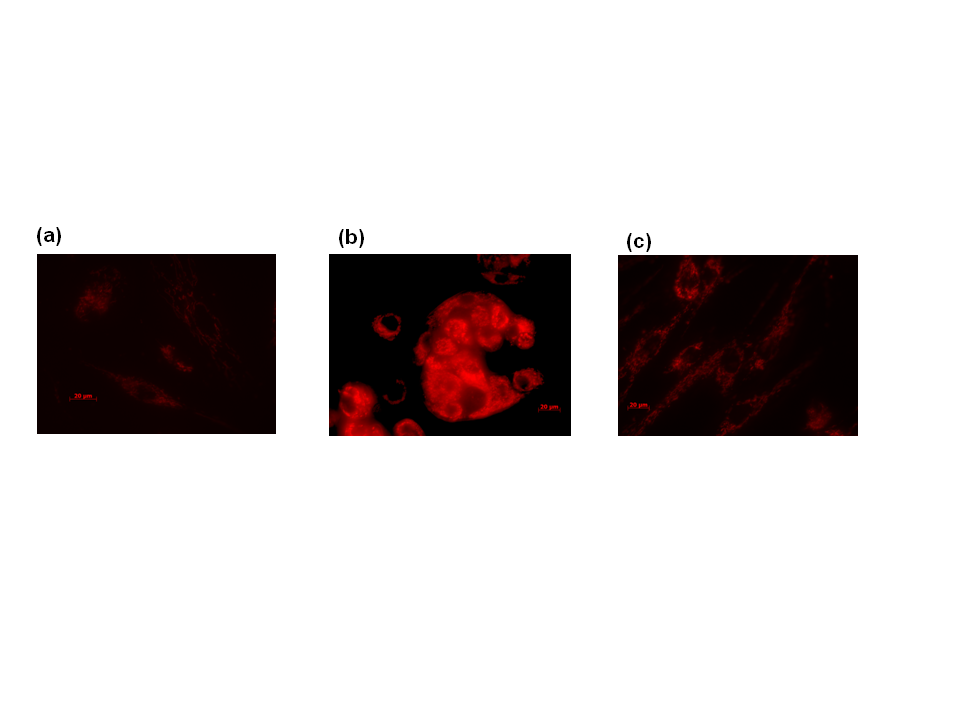

Supplement: Figure S1 — Fluorescence microscopy images of cryptphen in (a) NHDF, (b) MCF-7 and (c) P14; cells incubation with 10 nM of cryptphen during 1 h. (TIF) [file pone.0027078.s001.tif]

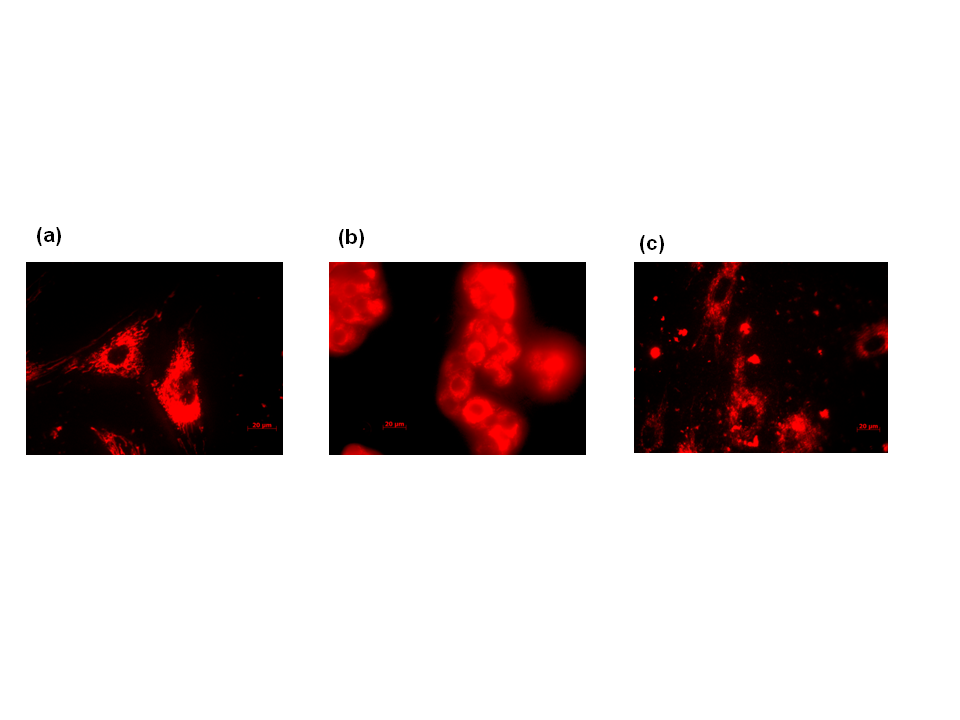

Supplement: Figure S2 — Fluorescence microscopy images of cryptphen in (a) NHDF, (b) MCF-7 and (c) P14; cells incubation with 10 nM of cryptphen during 3 h. (TIF) [file pone.0027078.s002.tif]

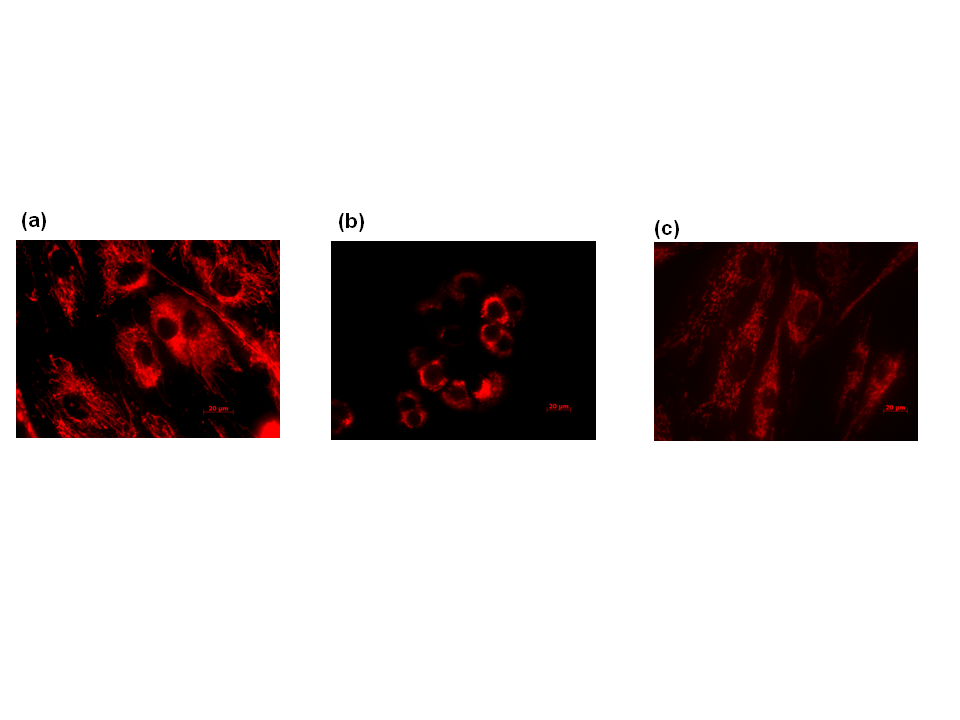

Supplement: Figure S3 — Fluorescence microscopy images of cryptphen in (a) NHDF, (b) MCF-7 and (c) P14; cells incubation with 10 nM of cryptphen during 6 h. (TIF) [file pone.0027078.s003.tif]

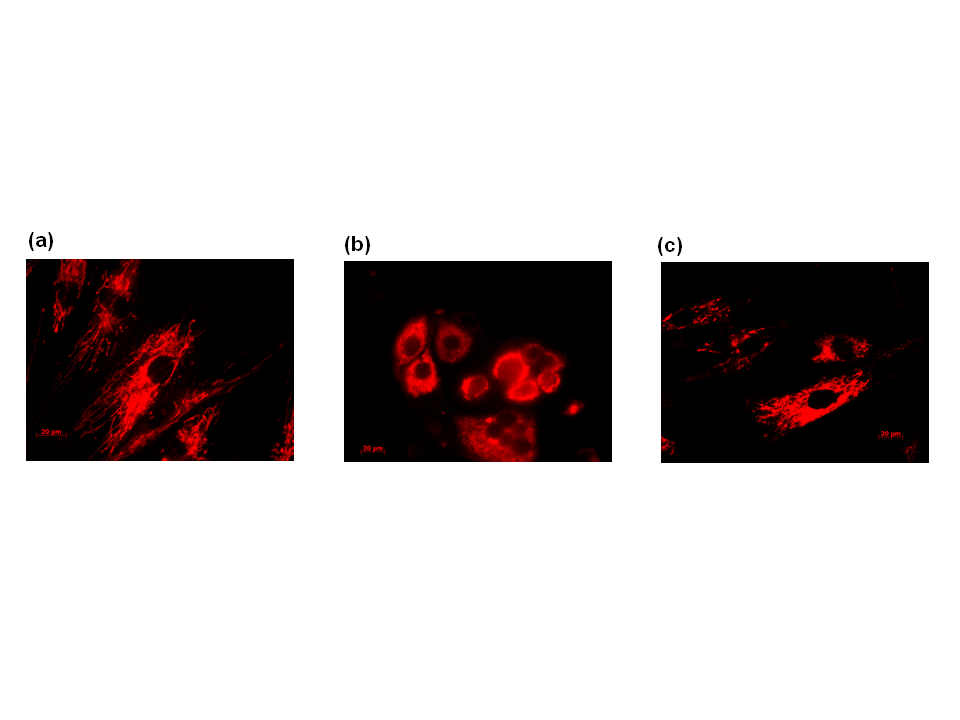

Supplement: Figure S4 — Fluorescence microscopy images of cryptphen in (a) NHDF, (b) MCF-7 and (c) P14; cells incubation with 10 nM of cryptphen during 24 h. (TIF) [file pone.0027078.s004.tif]

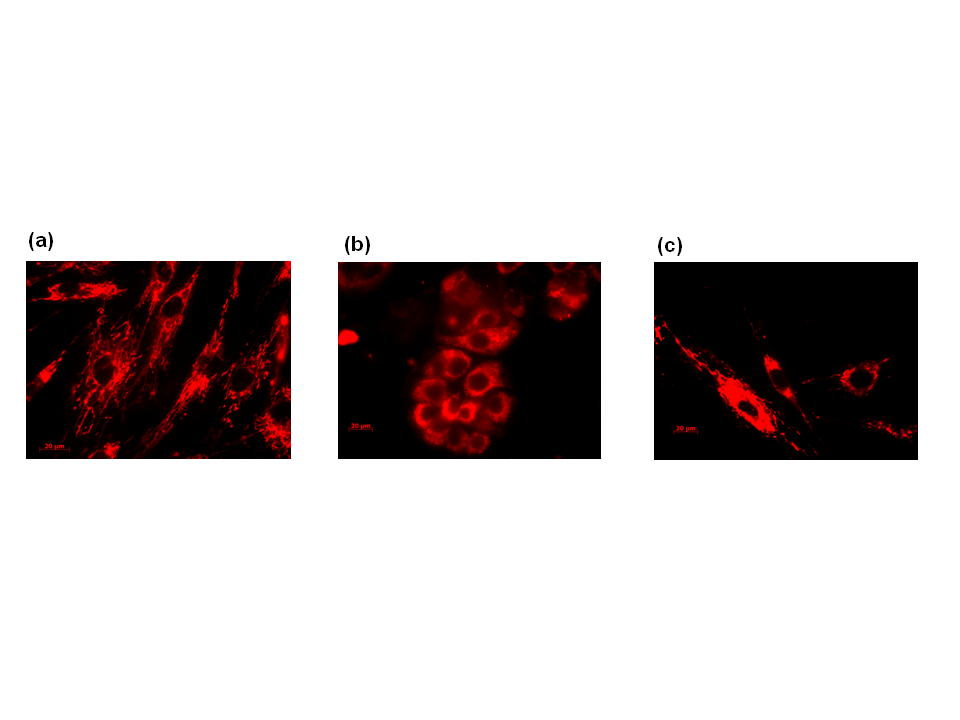

Supplement: Figure S5 — Fluorescence microscopy images of cryptphen in (a) NHDF, (b) MCF-7 and (c) P14; cells incubation with 10 nM of cryptphen during 36 h. (TIF) [file pone.0027078.s005.tif]

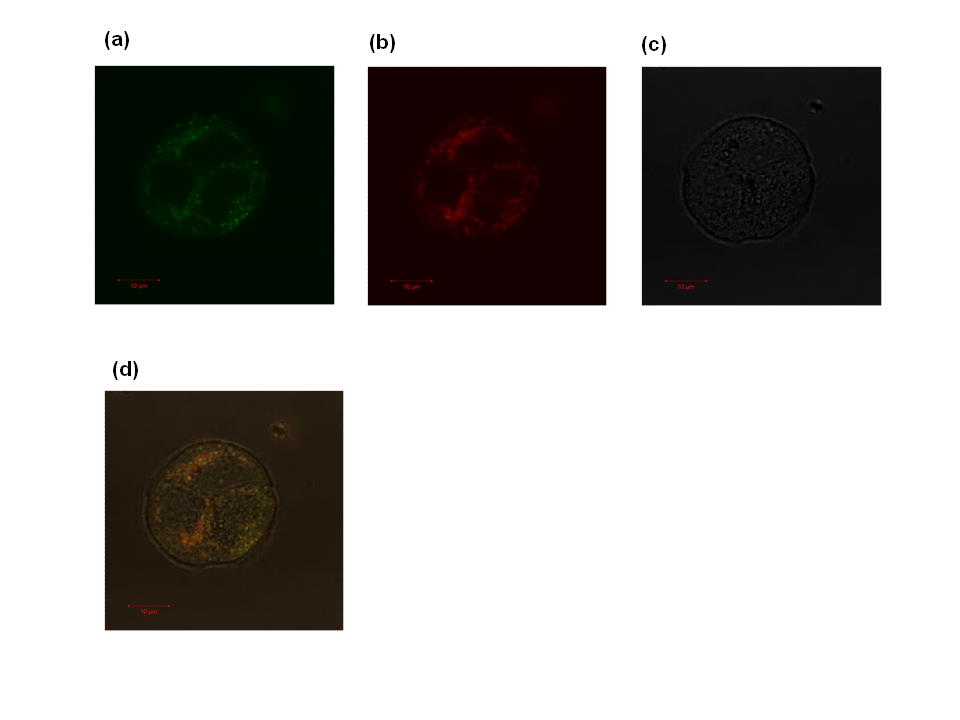

Supplement: Figure S6 — Single laser scanning confocal microscopy images of MCF-7 cells (1×10^4^ cells/dish) treated with 2,4-DNP (300 µM, 2 h) and stained with [30]phen2N6 (100 nM, 1 h) and MitoTracker Green™ (25 nM, incubation time 30 min); (a) fluorescence images of MitoTracker Green™, (b) [30]phen2N6, (c) differential interference contrast (d) and merged images of red and green channels; scale bar 10 microns. (TIF) [file pone.0027078.s006.tif]

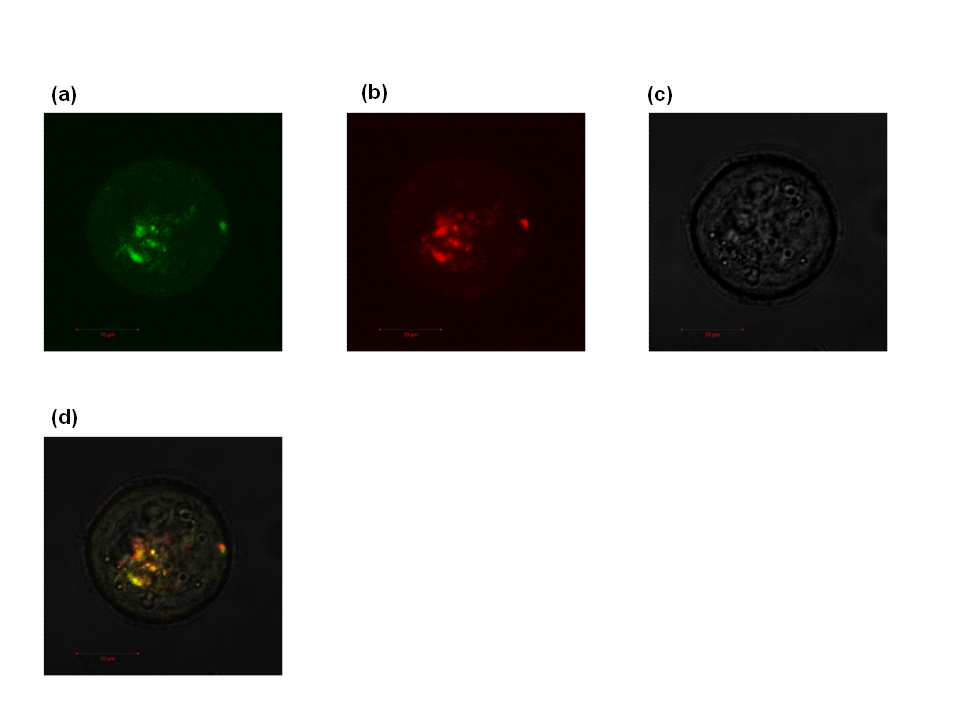

Supplement: Figure S7 — Single laser scanning confocal microscopy images of MCF-7 cells (1×10^4^ cells/dish) stained with [30]phen2N6 (100 nM, 1 h) and MitoTracker Green™ (25 nM, incubation time 30 min) without 2,4-DNP; (a) fluorescence images of MitoTracker Green™, (b) [30]phen2N6, (c) differential interference contrast (d) and merged images of red and green channels; scale bar 10 microns. (TIF) [file pone.0027078.s007.tif]
